# The Expanding Role of Nurse Practitioners in the Completion of Oregon Portable Orders for Life-Sustaining Treatment

**DOI:** 10.1089/jpm.2021.0386

**Published:** 2021-09-20

**Authors:** Susan W. Tolle, Abby Dotson, Betty Ferrell

**Affiliations:** ^1^ Division of General Internal Medicine and Geriatrics, Center for Ethics in Health Care, Oregon Health and Science University, Portland, Oregon, USA.; ^2^ Emergency Medicine, Oregon Health and Science University, Portland, Oregon, USA.; ^3^ Division of Nursing Research and Education, Department of Population Sciences, Beckman Research Institute of City of Hope, Duarte, California, USA.

*Dear Editor:*

## Introduction

**FIG. 1.**
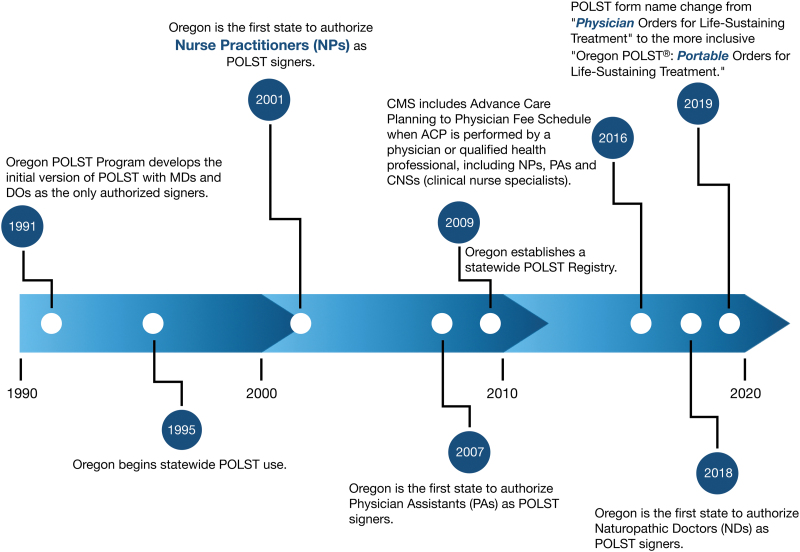
Oregon POLST program's history of becoming more inclusive. ACP, advance care planning; CMS, Centers for Medicare and Medicaid; DOs, doctors of osteopathic medicine; MDs, medical doctors; POLST, Portable Orders for Life-Sustaining Treatment.

Oregon established the Portable Orders for Life-Sustaining Treatment (POLST) program to honor treatment wishes of patients with advanced illness and frailty as they moved across care settings.^1^ At the time POLST was initiated, the scope of practice for nurse practitioners (NPs) and physician assistants (PAs) was limited and only medical doctor (MD) and doctor of osteopathic medicine (DO) licensees were authorized signers (Fig. 1). Oregon began statewide POLST use in 1995 and prospectively collected data documenting a 5% in hospital death rate for nursing home residents with orders for Do Not Resuscitate (DNR) and Comfort Measures Only. NPs became authorized signers in Oregon in 2001. Although research about POLST has proliferated, little attention has been given to the growing role of NP signers.^2,3^ We report in this study our ongoing research on the role of NPs in completion of POLST forms and review Oregon's history of becoming more inclusive.

## Results

This study included 121,956 POLST forms that were entered into the Oregon POLST Registry between January 1, 2018 and December 31, 2020, and were signed by one of five disciplines: MD, DO, NP, PA, and naturopathic doctor (ND).^4^ Of these 22,384 (18.4%) were signed by an NP, whereas 79,796 (65.4%) were signed by an MD, 13,382 (11.0%) by a DO and 6295 (5.2%) by a PA. NDs signed 0.1%. Each year the percentage of POLST forms signed by NPs increased. In 2018 the rate was 16.8%, in 2019 it was 17.9%, and in 2020 the rate rose to 21.0%.

When adjustments are made for this threefold difference in the number of licensees, MDs, and NPs complete POLST forms with the same frequency. In 2020, 15,687 MDs completed 21,465 POLST forms for a rate of 1.37 forms per active licensee, whereas 5186 NPs completed 7236 POLST forms for a rate of 1.4 forms per active licensee. NPs signed 9.0% of forms in 2010 and 11.9% of forms in 2015 with an average rate of 10.9% over the 2010 to 2015 interval. This compares with the rate of 21.0% in 2020, doubling the rate that NPs sign POLST forms for the past decade. This change occurred during a time when the number of NP licensees rose 109%, whereas the number of MDs increased by 49% and Oregon's population increased 11%.

## Discussion

The vital role NP and PA colleagues serve in caring for patients near the end of life was recognized by the Centers for Medicare and Medicaid Services in 2016, when Advanced Practice Registered Nurse (APRN) and PA professionals were included in the advance care planning fee schedule.^5^ Studies from Oregon and West Virginia document the growing role of NPs in POLST form completion.^2,3^ The Oregon POLST Coalition recognized these vital contributions and changed the name of POLST to be more inclusive replacing the word “physician” with the word “portable.” On January 1, 2019, Oregon POLST became known as Portable Orders for Life-Sustaining Treatment.

## Conclusions

NPs are currently completing one fifth of all POLST forms entered into the Oregon POLST Registry, suggesting more inclusive interdisciplinary involvement in health care decisions.
